# Optimal Subset Selection of Time-Series MODIS Images and Sample Data Transfer with Random Forests for Supervised Classification Modelling

**DOI:** 10.3390/s16111783

**Published:** 2016-10-25

**Authors:** Fuqun Zhou, Aining Zhang

**Affiliations:** Canada Centre for Remote Sensing, Natural Resources Canada, 560 Rochester Street, 6th Floor, Ottawa, ON K1A 0E4, Canada; Aining.Zhang@canada.ca

**Keywords:** time-series Moderate Resolution Imaging Spectroradiometer (MODIS), Random Forests, data mining, supervised classification modelling, land cover classification, sample transferability

## Abstract

Nowadays, various time-series Earth Observation data with multiple bands are freely available, such as Moderate Resolution Imaging Spectroradiometer (MODIS) datasets including 8-day composites from NASA, and 10-day composites from the Canada Centre for Remote Sensing (CCRS). It is challenging to efficiently use these time-series MODIS datasets for long-term environmental monitoring due to their vast volume and information redundancy. This challenge will be greater when Sentinel 2–3 data become available. Another challenge that researchers face is the lack of in-situ data for supervised modelling, especially for time-series data analysis. In this study, we attempt to tackle the two important issues with a case study of land cover mapping using CCRS 10-day MODIS composites with the help of Random Forests’ features: variable importance, outlier identification. The variable importance feature is used to analyze and select optimal subsets of time-series MODIS imagery for efficient land cover mapping, and the outlier identification feature is utilized for transferring sample data available from one year to an adjacent year for supervised classification modelling. The results of the case study of agricultural land cover classification at a regional scale show that using only about a half of the variables we can achieve land cover classification accuracy close to that generated using the full dataset. The proposed simple but effective solution of sample transferring could make supervised modelling possible for applications lacking sample data.

## 1. Introduction

Land cover mapping at a regional scale provides essential information for monitoring and dynamic assessment of the environment, as well as for economic and social impact assessment [1]. Moderate-Resolution Imaging Spectroradiometer (MODIS) Earth Observation data with daily coverage, multi-bands and moderate spatial resolution are suitable datasets for such applications. Numerous studies have shown promising outputs using MODIS data in land cover classification at large scales. For example, Friedl et al. [2] have used MODIS data for global land cover mapping, and Knight et al. [3] characterized regional scale land cover with MODIS imagery. MODIS data have also been widely used for agricultural land cover mapping and land surface information retrieval. Wardlow and Egbert [4] mapped large-area crop with MODIS Normalized Difference Vegetation Index (NDVI); Alcantara et al. [5] conducted abandoned agriculture mapping using MODIS satellite data; Guindin-Garcia et al. [6] evaluated MODIS 8- and 16-day composite products for maize green leaf area index monitoring; and Xiao et al. [7] proposed a framework for consistent estimation of multiple land surface variables such as leaf area index and surface albedo etc. from MODIS time-series data. Among these studies, time-series or multi-temporal MODIS data are utilized. These studies indicated that time-series MODIS data are valuable information sources for environmental studies. Currently, various time-series MODIS datasets are freely available, for example, MODIS 8- and 16-day composites with global coverage from NASA [8,9], and 10-day clear-sky MODIS composites with the coverage of Canadian landmass from the Canada Centre for Remote Sensing (CCRS) [10]. These time-series MODIS datasets contain rich information of seasonal dynamics of land surface phenomena; however, using these time-series MODIS datasets poses some challenges to applications due to their vast data volume and information redundancy in regards to efficient and effective information retrieval. For example, CCRS 10-day clear-sky MODIS composite product has 36 composites per year; each of them has seven bands. Using all the data together may not be an optimal way to realize the benefits of seasonal information captured by the time-series MODIS dataset; however, research on how to efficiently use this kind of data is limited. In recent years, only a few studies have explored this issue: Zhou et al. [11] explored optimal variable combination and derived information such as NDVI and phenology of time-series MODIS data with See5/C5 for land cover mapping, and Nitze et al. [12] studied temporal optimization of image acquisition for land cover classification with Random Forests and MODIS time-series. These studies showed that optimal data utilization would remove information redundancy, increase computation efficiency, and at the same time reach the highest possible classification accuracy when full datasets are used.

Random Forests (RF) [13] has been broadly used by the remote sensing community for Earth Observation data applications [12,14,15,16,17,18,19]. These studies demonstrated that RF is a useful tool for information retrieval and land cover classification of remotely sensed data. In addition, Random Forests has many valuable features such as variable importance in modelling and outlier identification for data quality control [19].

Random Forests is an ensemble of decision trees. Variable importance of RF is a score which represents the variable’s contribution to the building of the decision trees. The bigger the importance score, the richer the information it contributes to the modelling, and vice versa. Random Forests gives an option of running a model using only those important variables for computation efficiency without compromising accuracy of the model output. In this study, we explored the RF’s variable importance feature to value the contribution of every band at different time frame of CCRS 10-day clear-sky time-series MODIS composites to land cover classification, and then analyzed the optimal subsets (bands and season combinations) for efficient usage of the dataset, which forms the major goal of the study.

Random Forests is capable of flagging outliers of sample data. The feature is beneficial to sample data screening for improving data quality in case some of the sample data collected for modelling are wrongly selected, or not representative of the data population. In this study, we used the outlier feature for another purpose. A supervised classification paradigm requires feature samples for model training and validation. Sampling exercise is usually expensive when dealing with mapping of a large region. In some cases, even no samples are available at a certain temporal period of a study. For example, for time-series analysis, especially for historical data analysis, it is common that researchers face a problem of lacking sample datasets so supervised classification modelling cannot be employed. In some situations, sample data are available at certain timeframes. In order to use supervised modelling approach of classification, we need to find a way to extrapolate data for those periods without samples using existing knowledge or samples from other time periods. This not only can solve the modelling problem, but also has large economic benefit. Hence, one of the objectives of the study is to explore the outlier feature of Random Forests to help adopt samples from one timeframe to an adjacent one for supervised modelling. The tackling of the two important and practical issues of this study is demonstrated by a case study of land cover classification over an agricultural region using CCRS 10-day clear-sky time-series of MODIS composites.

## 2. Study Region and Datasets

### 2.1. Study Region

**Figure 1:**
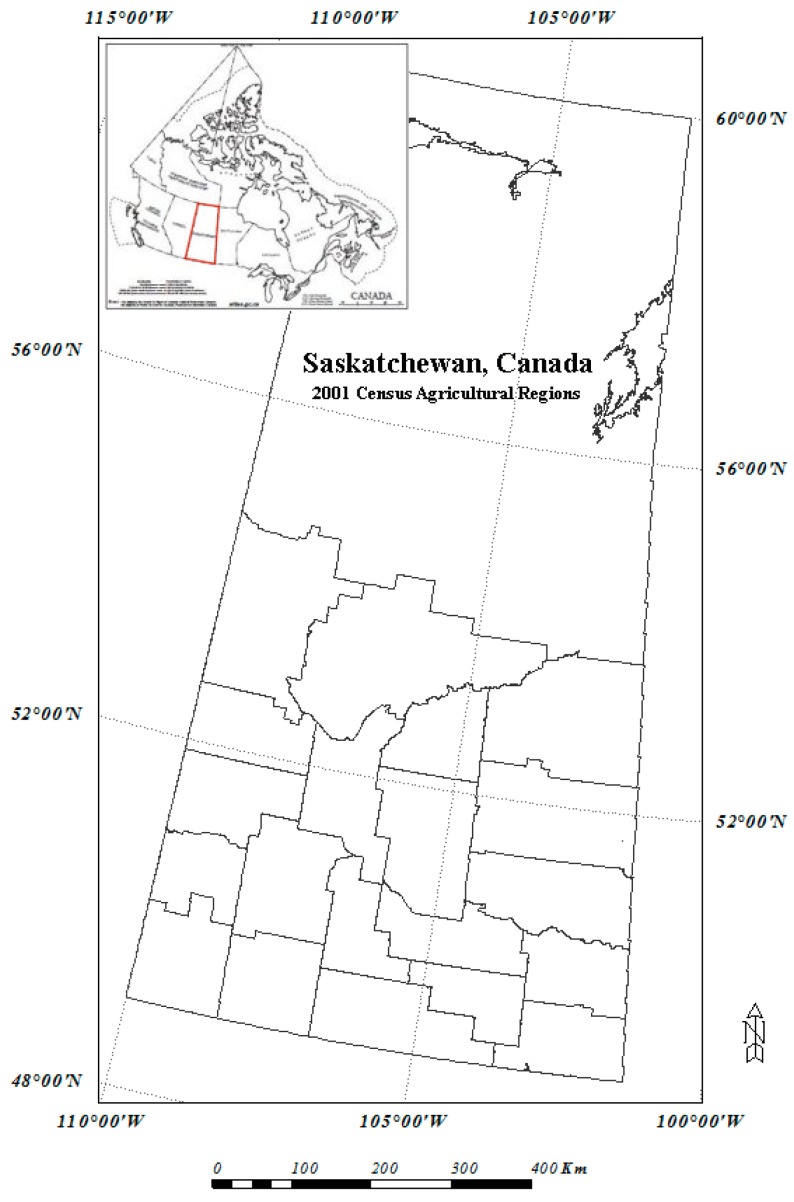
The study region—southern Saskatchewan, Canada. The polygons are Census Agricultural Regions (CARs).

The region of interest in this study is southern Saskatchewan Province, Canada, shown in Figure 1. Saskatchewan is part of the Western Provinces of Canada and designated a Prairie region. It is the only province of Canada with entirely artificial boundaries. It is bordered by the United States to the south, the Northwest Territories to the north, and Manitoba and Alberta to the east and west, respectively.

Saskatchewan contains two major natural regions: the Canadian Shield in the north and the Interior Plains in the south. Northern Saskatchewan is mostly covered by boreal forest except for the Lake Athabasca Sand Dunes, the largest active sand dunes in the world north of 58°. Southern Saskatchewan contains another area with sand dunes known as the “Great Sand Hills” covering over 300 km^2^. The Cypress Hills, located in the southwestern corner of Saskatchewan and Killdeer Badlands (Grasslands National Park), are areas of the province that remained unglaciated during the last glaciation period.

The southern part of the province is the area level or gently rolling plains marked by fertile soils that make Saskatchewan one of the world’s greatest wheat producers. Saskatchewan is vast, with an area about 651,000 km^2^. It has almost half of Canada’s total cultivated farmland, and commonly called the “grain belt” [20]. Thus, it is one of the best places for studying agricultural land cover mapping using remotely sensed data.

### 2.2. Earth Observation Data

The Earth Observation (EO) data used in the study is CCRS 10-day clear-sky time-series MODIS composites, which are developed from the original Terra MODIS swaths. On top of the cloud screening processing for 10-day composition, the dataset is resampled to 250 m spatial resolution for bands 3 to 7 and reprojected to Lambert Conformal Conic (LCC) to better fit to the geographic region of Canada [10]. The last two features of the dataset are the main reasons for its preference to other MODIS time-series datasets as the EO data input to the study. As the result of the 10-day compositing, there are a total of 3 composites per month. The agricultural production of the region under the study has only one growing season, so only the composites in the growing season, i.e., from April to October, are used in the study. Hence, the time-series MODIS dataset utilized in the study consists of 21 composites and seven bands for each composite. In total, there are 147 variables (7 bands × 21 composites).

Although CCRS 10-day MODIS composites are called clear-sky, there are still some cloud and haze residues on the data. Before the data are used, a median rank filter of five elements in the time dimension is applied to the dataset to remove residual spikes [21]. The output of the rank filter on the original 10-day composites is then the input to the land cover classification modelling and analysis.

Two years (2000 and 2001) CCRS time-series MODIS data are used in the study. The dataset of year 2000 is mainly used for model evaluation and analysis of optimal subset selection with the help of RF variable importance feature. The dataset of year 2001 is used to examine the efficiency of sample transferring from 2000 to 2001 for supervised classification modelling by using RF’s outlier identification feature. This is demonstrated by the comparison of the generated 2001 agricultural land cover classification with the census data of 2001 Census of Agriculture over the study region.

### 2.3. Land Cover for Agriculture Regions of Canada, Circa 2000

Circa 2000 land cover map for agricultural regions of Canada is a 30 m resolution raster format of land cover classification (alternatively called 30 m Circa 2000) produced by Agriculture and Agri-Food Canada (AAFC) mainly from Landsat imagery [22]. The map covers all agricultural regions across Canada’s 10 provinces with the goal of accurately classifying lands at a moderate scale that are annually cultivated (cropland), permanent crop and pasture, native grassland, as well as other contextual classes (such as built-up and forested lands). The purpose of the mapping is to provide information that can be used to support land use decision making and a range of other federal, provincial, municipal and private agri-environmental projects, applications, programs and activities [22]. To date, the data is the most recent and has the highest resolution of land cover map covering the entire agricultural regions of Canada. The Circa 2000 land cover map is of considerable accuracy for use as a reference dataset. Its positional accuracy is within 1 pixel (30 m). A cross-validation measure based on training data of each of the 97 scenes demonstrated an overall accuracy of 86.6%. A thematic consistency measurement of 151 overlapping cases resulted in an average percentage consistency of 93.1%.

The Circa 2000 land cover map has 11 major land cover types: Annual cropland, Perennial cropland and pasture, Native grassland, Shrubland, Forest land (Coniferous, Deciduous, and mixed), Water body, Developed land, Wetland, Exposed land, as well as an unclassified land. As the MODIS dataset used in the study is of 250 m spatial resolution, the much higher spatial resolution (30 m) of the Circa 2000 map is used as a reference dataset of the study. To be geographically matched to the MODIS dataset, Circa 2000 land cover map is reprojected to Lambert Conformal Conic and resampled to 250 m. This processing produced a land cover map of the region in 250 m (alternatively called 250 m Circa 2000). Companion to the resampled land cover map, a homogeneity indicator map covering the same region is generated. The indicator map has three values of 0, 1, and 2. Value 2 indicates that the pixel of 250 m Circa 2000 is homogeneous in the sense that all the original corresponding subpixels (in total, about 64 subpixels) of the 30 m Circa 2000 have the same land cover type; Value 1 indicates that the pixel of the 250 m Circa 2000 is a dominant one, which means that a land cover type occupies at least half of its original subpixels; Value 0 represents a heterogeneous pixel within which no land cover type is dominant. The indicator map is essential for supporting selection of training and validation samples from 250 m Circa 2000 for the classification modelling with CCRS MODIS data and Random Forests. In this study, only homogeneous pixels are selected for model training, as well as for model validation.

### 2.4. 2001 Census of Agriculture and Census Agricultural Region

Statistics Canada conducts a survey of agricultural industry across Canada every five years (e.g., 1996, 2001, 2006, etc.). The Census of Agriculture collects a wide range of data on the agriculture industry including land use for crops, grassland and forest lands etc. For gathering the information, Statistics Canada sends a questionnaire to farm operators, and then analyzes and synthesizes the responses [23]. Synthesized data of the Census of Agriculture are based on different levels of geographic regions of a province: Census Consolidated Subdivision, Census Divisions, and Census Agricultural Regions (CARs). The base unit of Census of Agriculture is Census Consolidated subdivision, followed by Census Division (CD), and Census Agricultural Region (CAR), then province with the exception that the boundaries of CDs and CARs are not overlapped in Saskatchewan. For this case study of land cover mapping at the regional level, CAR is used as the geographic base unit. The boundaries of CARs of the study region (southern Saskatchewan) are shown in Figure 1. There are 20 CARs for 2001 Census of Agriculture of the study region. The original CARs are polygon-based which are rasterized to 250 m × 250 m grids to match to the spatial resolution of the MODIS imagery. The gridded CAR layer is used for statistical analysis of land cover type information within CARs.

One of the reasons of using 2001 Census of Agriculture for the study is that in-situ sample data of land cover over the region in 2001 is not available; however, Circa 2000 land cover map of the same region is available. Therefore, this is a practical case for investigating the usefulness of adapting sampling datasets of 2000 to 2001 for supervised land cover classification modelling. In addition, there are 20 CARs of Census of Agriculture over the study region. These datasets act as a “ground truth” for us to check the spatial behavior of the modelling in addition to Kappa coefficient for accuracy assessment. As the survey is conducted only once every five years, the land cover/use information is lacking between those census years. The survey is costly and time consuming, and survey results are only available about one year after the survey. This study could provide some insights if the proposed method will generate equivalent information such as cropland acreage between survey years and fill the information gap in a cost effective and time efficient manner.

## 3. Methodology

### 3.1. Classification Model

Random Forests is an ensemble of decision trees, and a random forest model builds many classification trees based on a training dataset. To classify a new object from an input data vector, the model puts the data down each of the trees in the forest and each tree gives a classification (vote). The forest chooses the classification having the most votes [19]. Random Forests is one of the highest performers among current algorithms in terms of classification accuracy, computation efficiency, and transparency. In this study, we go beyond only using Random Forests’ capability of classification modelling. The variable importance and outlier identification features of Random Forests are investigated for choice of optimal subsets and transferring of sample data for supervised land cover classification modelling with CCRS 10-day clear-sky time-series MODIS composites.

The land cover classification system adopted in this study is the one from Circa 2000 land cover for agriculture regions. In this study, we consider the main 9 land cover types: Annual crop, Perennial crops and pasture, Native grassland, Water, Developed land, Coniferous forest, Deciduous forest, Mixed forest, and Shrubland.

### 3.2. Sampling Strategy

Like a classic supervised classification model, the supervised RF model requires a set of training data and another set of sample data for validation of the model developed. For the relatively large region (~651,000 km^2^) of the study using 250 m spatial resolution of MODIS data, Circa 2000 land cover map is suitable as a reference dataset for model training and validation since it is produced from much higher spatial resolution of Landsat data and covers the entire study region.

The procedures of selecting sample data from the reference map are as follows: first, the study area is divided into 20 equal sub-regions (5 by 4) which roughly represent various geographic situations at different locations in the region. Then the sampling exercise is carried out within every sub-region for all the land cover types to be mapped: two sets of 200 samples for each land cover type are randomly drawn from every sub-region if the land cover indicator map shows that the pixel at the location is homogeneous; one set is used for model training, and the other is for model validation. Summed from all the sub-regions, there are 4000 samples in total for each land cover type. The sampling process is to guarantee that all land cover types over the entire region are equally represented.

Corresponding to the land cover training samples, the spectral signatures of CCRS time-series MODIS imagery of years 2000 and 2001 at the sampling locations are extracted. The samples of year 2000 are directly used for RF modelling and validation while the samples of year 2001 need an extra screening process in order to be used for 2001 classification modelling which is described below.

### 3.3. Sample Data Transferring

A common problem in dealing with time-series of imagery is that training and validation samples are only available at some years but unavailable for other years. An example of this is yearly land cover change detection by using supervised classification approaches. Using the knowledge or information at one point in time to other times without knowledge is broadly known as transfer learning. Transfer learning basically describes the processes of retaining and applying the knowledge available for one or more tasks, domains, or distributions to efficiently develop an effective hypothesis for a new task, domain, or distribution. Instead of involving generalization across problem instances, transfer learning emphasizes the transfer of knowledge across tasks, domains, and distributions that are similar but not the same [24]. There are several domain adaptation techniques such as the algorithms treating source-domain data as prior knowledge and estimating the target-domain model parameters under such prior distribution. For example, Hwa [25] and Gildea [26] demonstrated that simple techniques based on using adequately selected subsets of source-domain data and parameter pruning can improve the performance on the target data.

In this study, we selected two adjacent years (2000 and 2001) for testing our proposed method of sample data transferring. Since we have a detailed reference map of year 2000, and none of year 2001, the sample data of the former are considered as source-domain and the latter is treated as target-domain. Although the available sample data from the source-domain could largely represent the target (2001) population due to relatively small changes of land cover from 2000 to 2001 for the study region, they might cause a poor estimation of the target distribution and introduce a bias in the estimated class prior distribution if used directly. In order to make supervised RF modelling workable on 2001 MODIS imagery, we dealt with the problem of sample data transferring by simply removing the bias or outliers of the source-domain samples and then retain the suitable samples for the modelling of the target-domain data. In this regard, we exercised this process by using Random Forests’ outstanding feature of outlier identification. An outlier in RF is a case whose proximities to all other cases of the population are small, or an outlier in class j is a case whose proximities to all other class j cases are small. Assuming all samples of 2000 correctly represent land cover types and if all land cover types didn’t change from 2000 to 2001 over the sample locations, in theory, the samples of 2001 from the 2000 sample locations are still valid for the study of 2001. However, if some land cover types are changed over certain sample locations from 2000 to 2001, the changed samples of 2000 would become outliers if they were used for the study of 2001. In this regard, Random Forests’ outlier feature is used to identify outliers for the sample transferring. More details about proximity and outlier score computation of Random Forests can be found in the reference [19].

**Figure 2:**
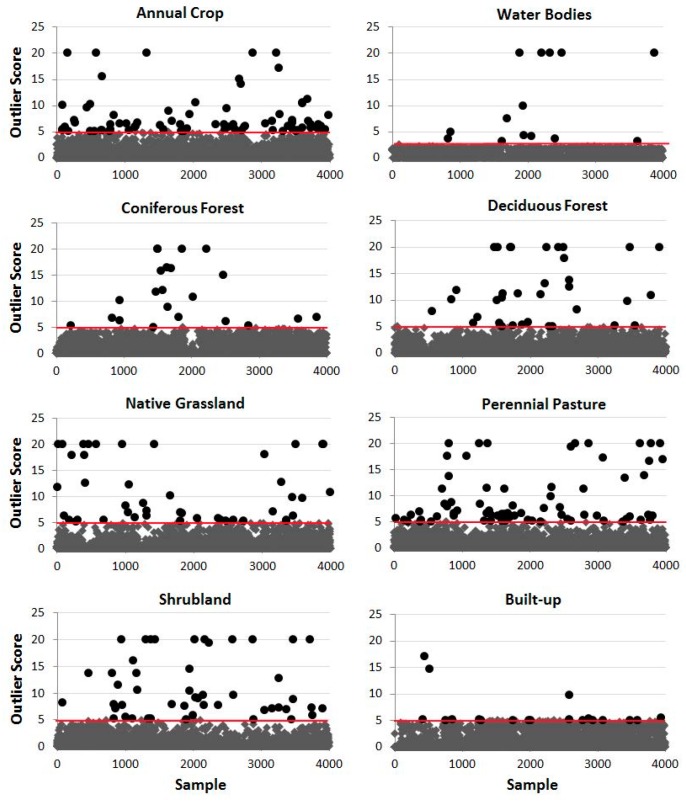
Random Forests’ outlier score distribution of year 2000 samples used for year 2001 modelling: Black circles: identified outliers; line in red (threshold): outliers are located above the line.

The rationale for transferring the samples of the Circa 2000 land cover map with exclusion of outlier samples to the study of year 2001 are based on the following considerations: the study region is dominated by agricultural production, so the chance of land cover type change on the sampling locations from one year to another is small. Furthermore, if the land cover type does change at some locations, it is expected the MODIS spectral signatures should change accordingly, and become different from those of other samples of the same land cover type, i.e., they should become outliers from the sample population, instead of clustering with other samples of the land cover type. In order to identify the sample pixels whose land cover type changed from the previous year and are no longer valid as training or validation samples, the RF’s outlier identification feature was applied to the sample data. For this purpose, two RF model runs are conducted. In the first run, all the samples are used for the RF modelling. The modelling process results in a classification model as well as an outlier score for every sample. A big outlier score means that the sample is remarkably away from the class that it is supposed to represent. Consequently, these outlier samples are removed from the sample datasets, and the remaining sample points are then used to develop a new RF model for the second run. Figure 2 shows the outlier score distribution of the samples of eight land cover types. It can be seen that the outlier scores of the majority of the samples are in the range of 0–5 for almost all land cover types and there are only a very small number of samples out of 4000 whose outlier score is greater than 5. The exception is Water body whose majority of outlier scores are under 3 and it has only about a handful samples whose outlier score is above 3. Based on these observations, in this study we used a threshold of 3 (outlier score > 3) for Water body and a threshold of 5 for all other land cover types to identify and remove outlier samples. With the threshold applied, less than 5% of the original samples were removed. After outlier removal, the size of the remaining samples for most of the land cover types was about 3800 out of the original 4000 samples. Therefore, the remaining samples are kept balanced.

**Figure 3:**
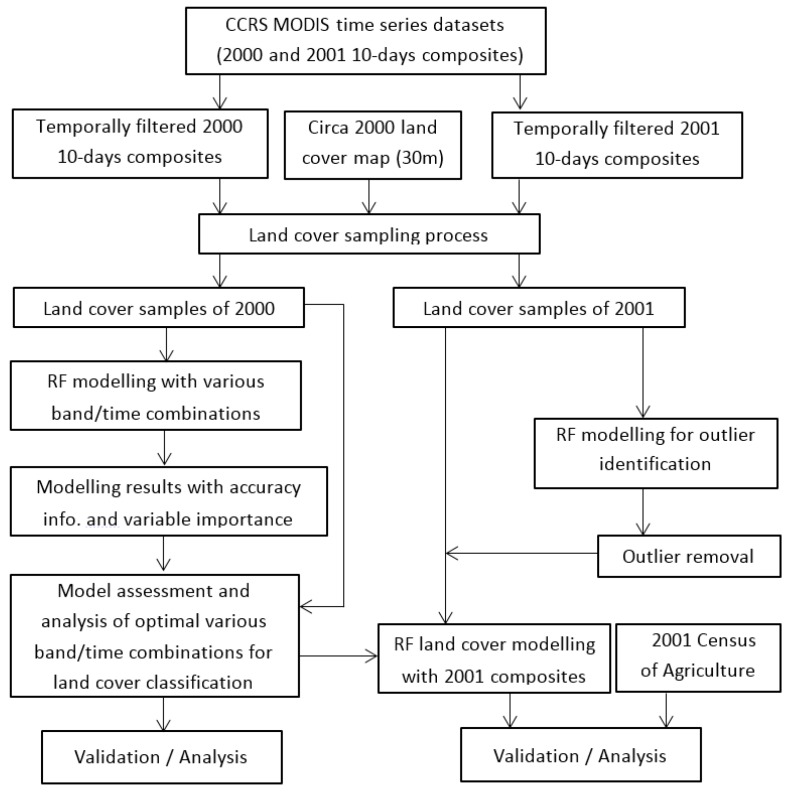
Overview outlining the key processing steps and datasets used in the analysis. CCRS: Canada Cetre for Remote Sensing; MODIS: Moderate Resolution Imaging Spectroradiometer; RF: Random Forests.

More discussion about the method for sample transferring and its advantages and shortcomings, as well as suggested further study on the issue can be found in Section 5.2. Due to the multiple datasets and processing steps involved in the analysis an overview of the analysis is provided in Figure 3. The top portion of Figure 3 displays the data used in the study (imagery and reference dataset); the left of the figure shows the procedures of RF modelling for year 2000, and the right of the figure represents the process of data transferring for RF modelling for year 2001.

## 4. Results and Analysis

### 4.1. RF Modelling and Its Validation

An RF model developed by the training samples and validated by the verification samples outputs classification results with various corresponding information with regards to the classification accuracy which includes Out of Bag (OOB) error and a confusion matrix of the classification. OOB error is an unbiased estimate of classification error. In random forests, each tree is constructed by using a different bootstrap sample set from the training data. About one-third of the cases are left out and not used in the construction of the *k*th tree. To get the OOB error, put each case left out down the *k*th tree to get a classification. In this way, a test set classification is obtained for each case in about one-third of the trees. At the end of the run, take *j* to be the class that got most of the votes every time case *n* was OOB. The proportion of times that *j* is not equal to the true class of *n* averaged over all cases is the OOB error estimate [19]. In the study, both OOB error and Kappa (whose value is derived from the confusion matrix) are used as indicators of the classification accuracy. At the same time, some analysis on misclassification between land cover types is also performed.

**Figure 4:**
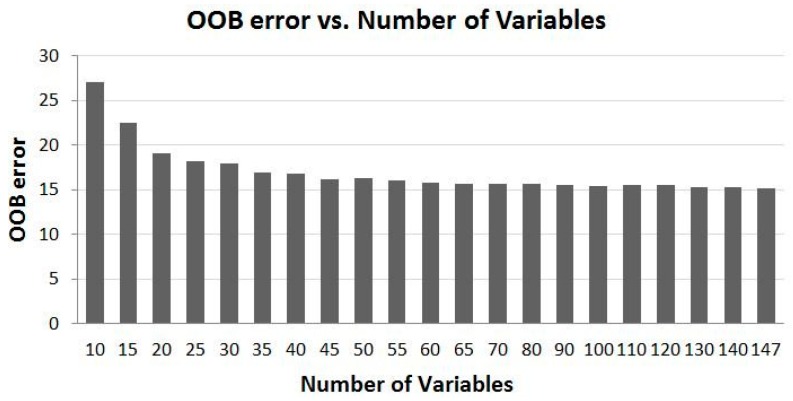
The relationship of Out of Bag (OOB) error and the number of the most important variables. OOB error decreases with the increase of the number of variables.

**Figure 5:**
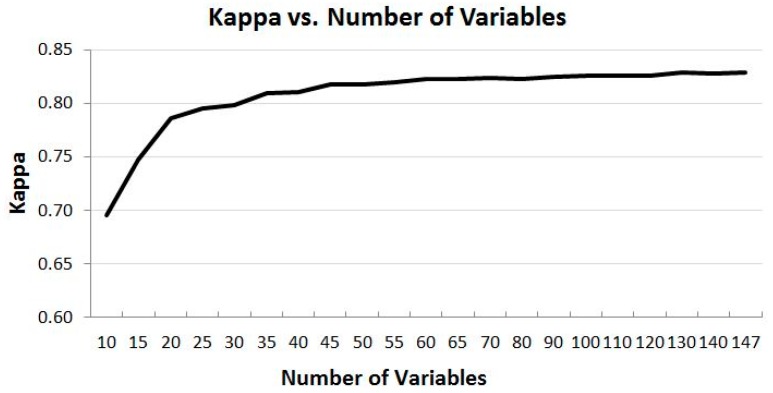
The relationship of Kappa and the number of the most important variables. Kappa increases with the increase of the number of variables.

The time-series 10-day cloud-free MODIS composites used in the study are from seven months from April to October. Within each month there are three composites and 21 bands, and each band is treated as a variable to the RF model input. With all the data (147 variables) used, OOB error is about 15% and Kappa is 0.829 for validation samples. These values indicate that the RF model performs reasonably well. To test the performance of the RF model with a reduced number of variables, about two dozens of runs with different number of variables from 10 to 140 in the order from the top of the highest importance scores are conducted. Figure 4 and Figure 5 show the relationships between the RF modelled OOB error, Kappa and the number of variables used in the modelling, respectively.

### 4.2. Overall Trend of Accuracy vs. Number of Variables

As shown in Figure 4 and Figure 5, it is obvious that time-series MODIS dataset improves the model output. OOB error decreases and Kappa increases as the number of variables increases, and vice versa. However, the relationship between them is not a linear one. The OOB error decreases and Kappa increases the most in the range of a smaller number of variables. For example, OOB error decreases about 5% from 27.04% to 22.46%, and Kappa increases about 0.05 from 0.696 to 0.747 when the number of variables increases from 10 to 15. However, the rate of change of OOB error and Kappa becomes smaller when the number of variables gets larger. For instance, when the number of variables increases from 60 to 70, the OOB error decreases only 0.05 from 15.76% to 15.71%, and Kappa increases by only 0.01. From these two figures, it can be concluded that increasing the number of variables does not vitally decrease OOB error and increase Kappa when the number of the variables reaches about 70, about half of the total number of the variables. This means that using a subset of the full dataset could achieve the almost the same accuracy as using all the variables.

### 4.3. Temporal and Band Frequency Analysis

With a RF classification output, the model generates a list of variable importance scores regarding its contribution to the modelling. Based on the multiple runs with various and reduced number of variables, we attempted to investigate the frequency of each band and each month and to explore if certain bands and months are more favorable in terms of their contributions to the land cover classification modelling.

#### 4.3.1. Variable Importance by Band

**Figure 6:**
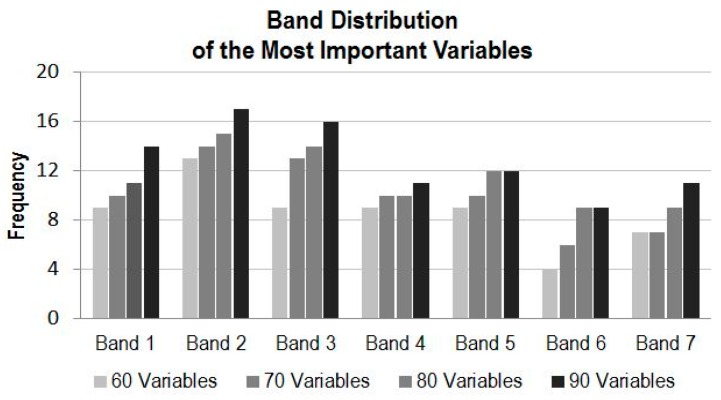
Band frequency with the number of the most important variables.

Figure 6 illustrates the band distributions involving 60, 70, 80, and 90 variables from the top of the variable importance score list. It can be seen that when only 60 variables are considered, Band 2 appears the most, followed by Band 1, Band 3, Band 4 and Band 5; however, the last four bands almost have the same frequency.

When the number of variables reaches 70, Band 2 still leads, but Band 3 gains more appearances while Bands 1, 4, and 5 increases at a similar but lesser pace. When the number of variables reaches 90, Band 2 still leads, followed by Band 3 then Band 1. In all cases, Band 6 is the least important whose frequency is just exceeded by Band 7. Figure 6 suggests Bands 1, 2, and 3 are the most important variables in terms of the land cover classification while Bands 6 and 7 are the least important when time-series data are used.

#### 4.3.2. Variable Importance by Time

**Figure 7:**
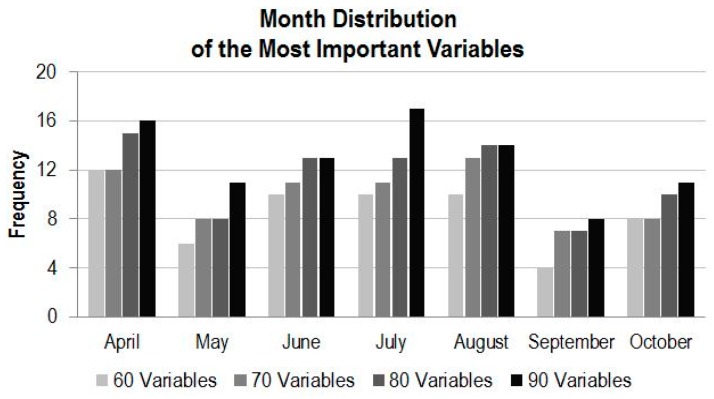
Month frequency with the number of the most importance variables.

The same variable importance scores for band frequency analysis are used for the frequency analysis in temporal dimension. Figure 7 shows monthly distributions of the most important variables in term of the classification modelling when a reduced number of variables are considered. When the first 60 most important variables are considered, image composites in April appear the most, followed, with almost equal frequency, by data in June, July and August. When the number of variables increases to 70, variables in August are more frequent than any other month, followed by the data in April, June and July. When the number of variables reaches 80, data in April is back on top, and when the number of variables increases to 90, data in July appear the most. Overall, data in April, June, July and August are more important (appear more frequently) than data in other months (May, September and October) based on the RF classification modelling and the variable contributions to the modelling. In all cases, data in May and in September contribute the least according to the RF modelling. In April, the grassland is greener than other vegetation types, which makes it more distinguishable from others. In May, the greenness of grassland and the cropland is likely similar, so they are hardly distinguishable. During the months of September or October, crops are harvested, so cropland is hardly distinguishable from grassland, which suggests that data acquired in September or October are the least informative for land cover classification in the study region. Overall, Figure 6 and Figure 7 reveal when different numbers of features are considered, the variable combinations from the time-series may be different. The figures also identify the variable combinations that produce better model performance.

### 4.4. Experimental Runs of the Selected Subsets of CCRS MODIS Dataset

Figure 4 and Figure 5 demonstrate possibility that a subset of the time-series MODIS data could deliver a similar result as using the full set of the MODIS data. Figure 6 and Figure 7 illustrate that the data from certain bands acquired in certain months are more favorable than others in terms of their classification capabilities. To verify the analysis presented above and to identify some optimal subsets for land cover classification practice, a few experimental runs with selected subsets of the time-series MODIS data are carried out. These subsets with a various number of variables are composed based on the information presented in Figure 6 and Figure 7. They consist of those data from four to six months and four to seven bands, whose total number of variables is from 72 to 108, which accounts for 49% to 73% of the original full time series of MODIS dataset, as listed in Table 1.

Based on Figure 7, the order list of month for subset data selection, from the most favorable to the least, is July, April, August, June, May, October and September. According to the list, if a subset of data is selected from only 4 months, data acquired in July, April, August, and June are preferable. If a subset of data is from five months, data from July, April, August, June as well as May can be selected, and so on. At the same time, the order list of band, based on Figure 6, is 2, 3, 1, 5, 4, 7 and 6. Similarly, if a subset of data is required only from four bands, the first four bands in the list, i.e., 2, 3, 1 and 5 are favorably selected, and so on. For the experimental runs, eight subsets with different number of variables combined from data in four to six months and from four to seven bands are composed (Table 1).

From Figure 4 and Figure 5, when the number of variables reaches about 70, the accuracy of the classification, as represented by Kappa and OOB error, does not differ much, but forms a plateau with increasing number of variables. When the number of variables is 70, Kappa is 0.823, and OOB error is 15.71%. These two numbers are used as the benchmarks for evaluation of the classification capability of a subset of data.

Table 1 shows the results of all the runs with possible optimal variable combinations. Compared to the benchmarks identified above, all the runs of the subsets of data from four to five months (subset data 1 to 5 in Table 1) yield a little poorer output, while the subsets of data from 6 months (subset data 6 to 8 in Table 1) produce a result equivalent to the benchmarks. It is noted that although the number of variables of all subsets from 4 to 5 months is greater than 70, their results are not as good as the benchmarks. It seems that they do not follow the pattern shown in Figure 4 and Figure 5 which reveal that when the number of the variables is more than 70, the accuracy should be equal to or a little greater than that of using 70 variables. However, Figure 4 and Figure 5 represent the best cases with the least number of variables and the best possible variable combination—its variables come from the entire band spectrum and all the months, i.e., information collected from the entire season is required although not all bands are utilized. The restriction of using the best possible variable combination is that land cover information can be retrieved only at the end of vegetation growing season as the variables are from the entire temporal period.

The accuracy of the last three runs (subset data 6 to 8 in Table 1) is equivalent to the benchmarks. The number of variables of these subsets varies from 72 to 108 whose variables are from four to six bands in six months. The increased accuracy, compared to those of the subsets (1 to 5), is due to usage of the variables from longer time series although the total number of variables is not larger. This further confirms that the length of time series data is a contributing factor to the improved accuracy of the land cover classification. According to the analysis above, for land cover mapping of the study region, an optimal subset with the highest possible accuracy output is those variable combinations from a longer time series of the dataset.

Some applications requires early season information, for example agricultural production prediction. In this regard, we run a test by using all the bands information from April to July (Subset data 9 in Table 1) to evaluate if the subset data in early season of the time-series data could provide the land cover information whose accuracy is close to that of the optimal subset with a longer time-series data. The run has a total of 84 variables from all the bands acquired in the first four months of the growing season, i.e., April to July. As expected, due to the shorter time period of the subset data, its accuracy indices (Kappa and OOB error) are worse than the benchmarks and that of any other subset data. However, the deviations from the benchmarks (Kappa 0.814 vs. 0.823; OOB error 16.525% vs. 15.71%) are relatively small. Hence, when the highest possible accuracy is not required, early season time-series of data could be used to substitute the optimal subset data for retrieval of early season.

### 4.5. RF Modelling for 2001 MODIS Data

After outlier removal using the method described above, the RF classification model has an OOB error of 16.69%, and Kappa of 0.812 based on validation samples. These values show that the model performs reasonable well although slightly poorer than those of the 2000 RF model outputs. The relatively poorer performance of the model is likely due to the training samples which are from the year 2000 even though outliers are excluded. With the output of the model, we generated a land cover classification map for year 2001 of the study region. We then overlaid the CAR polygons (Figure 1) to the land cover map, and extracted the occurrence of cropland, native grassland, as well as perennial crop and pasture within every CAR within the study region.

**Figure 8:**
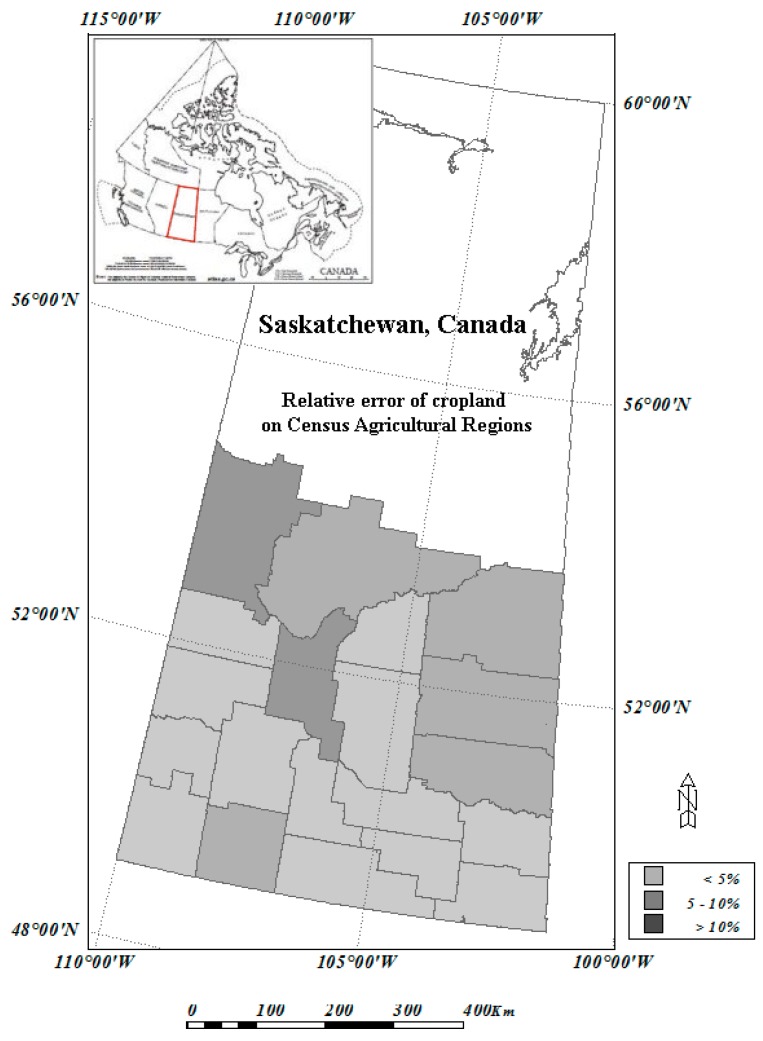
Relative error of cropland on Census Agricultural Regions (CARs): comparison between RF/MODIS result with the data of 2001 Census of Agriculture.

As shown in Figure 8, there are 20 CARs in the study region. The various gray shades represent the relative errors of the model output against the census data. For cropland, among the 20 CARs, 14 CARs have a relative error under 5%, and four CARs have a relative error between 5% and 10%, and only two CARs have a relative error greater than 10%. The majority of the CARs that have a larger relative error are located in the north of the study region, where the cropland area is relatively small; hence mathematically it is easier to have a larger relative error for those regions.

**Figure 9:**
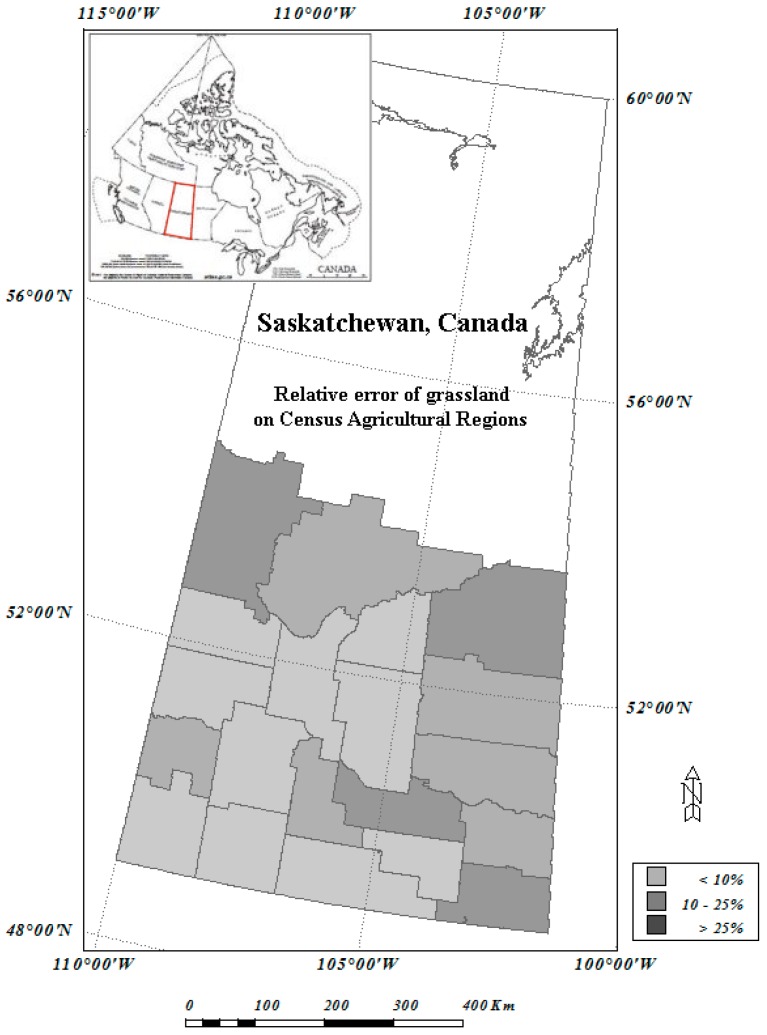
Relative error of grassland over CARs: Comparison between RF/MODIS result with the data of 2001 Census of Agriculture.

Information of two grassland land cover types are collected by the Census of Agriculture: one is tamed grassland and the other is natural grassland. The closest land cover types of Circa 2000 land cover map to the two land cover types are perennial crop/pasture, and native grassland. Since the definitions of the two grassland types of the two classification systems are not identical, they are grouped as one land cover type Grassland for the assessment. Figure 9 shows the relative error distribution of the Grassland produced by the modelling against the data of Census of Agriculture on CARs.

It can be seen that for the majority of CARs the data generated by the RF model resembles those of the census data, although Grassland does not match as well as cropland. Among the 20 CARs, half of them have a relative error under 10%; 6 CARs have a relative error between 10% and 25%; and the rest has a relative error over 25%. The relatively poor mismatch between the two datasets is likely due to relatively weak classification performance on Grassland. Grassland classification from remotely sensed data is usually poorer than other land cover types which are often reported [27,28]. Native grassland grows naturally, but in most of the cases, graze is one of the reasons to change its natural growing patterns. Perennial grassland usually grows with some human interruptions—it is likely harvested when it becomes mature, and it could grow again after harvest during the growing season, which is a part of the reasons that identifying grassland is harder compared to other land cover types. Another possible cause of the difference is due to the different land cover classification systems used for the comparison.

## 5. Discussion

### 5.1. Optimal Subset Data Selection

In this study, the optimal subset selection of MODIS time-series data for land cover mapping is based on Random Forests’ variable importance score, which is a measure of an input variable’s correctness count when it is classified with all the classification trees. As the variable importance is grounded on the statistics of the input data for modelling, the information generated from this study is of regional significance. Due to diversity of climates and landscapes, leading to different vegetation phenology over various regions, the output of this study may not be directly applicable to other regions or applications with different time-series EO data. For example, Nitze et al. [12] suggested that data in January and December are the best months to classify land cover types in their study; however, in Canada, there is only one growing season, and the data from January and December are not good choice for land cover mapping of Canadian agricultural regions.

It is obvious that optimal subset data selection from time-series Earth Observation data is a local issue. Therefore, the question should be examined with care according to the study region and application at hand. This study proposes a general method to identify optimal subsets using Random Forests tool, which can be broadly applied to other time-series EO data analysis over other regions. Once the optimal subsets are identified for an application, the knowledge can be used for long-term efficient monitoring of the study region such as for yearly land cover mapping.

The results of the optimal subset selection from this study are comparable to those of the study [11] using See5/C5 data mining software [29]. Both studies found that although a longer time-series EO data could produce a higher accuracy of land cover classification, the accuracy improvement with increasing the number of variables is not substantial when the number of variables reaches the plateau. Both studies identified Band 1 and Band 2 as important variables. One reason is that the spectral information of red (Band 1) and near infrared (Band 2) is useful for distinguishing vegetation from other features such as water and urban areas. Another reason is that the two bands have a higher spatial resolution than other bands, so they capture more detailed information for better discrimination of land cover types; however, there are some discrepancies from these two studies. For example, Band 6 is identified as the third important band for an optimal subset selection in the previous study using See5/C5, but it is considered as the least important band from this study. The difference likely comes from the different mechanisms how these two data mining tools use the time-series information of the dataset. Nevertheless, both See5/C5 and Random Forests are good data mining tools for information extraction from a large volume of data as the two tools produce similar accuracy of land cover classification using only an optimal subset of the time-series MODIS data.

### 5.2. Knowledge Transfer for Supervised Modelling of Classification

It is critical to have representative samples for supervised modelling of classification. There are cases when training and validation data are unavailable which prevent supervised modelling, such as lack of funding for field work or for historical data analysis. In this study, we proposed a simple but practical method of knowledge transfer of sample data from one year to an adjacent year for supervised classification. The advantage of this method is its simplicity and effectiveness. It takes full advantage of Random Forests’ robust computation of sample outlier score and can effectively identify and remove changed samples using an outlier threshold for knowledge transfer.

Although the method is considered practical and efficient based on this study, a more thorough study on the sample data transferring using RF’s outlier score of samples is needed. First, RF’s outlier score is inversely proportional to one case’s proximity to the case population. In general, a smaller outlier value of a case means it is closer to the population, and vice versa. Therefore, using a bigger outlier threshold would mark fewer outliers, while a smaller threshold would discriminate more outlier samples. Determining the optimal threshold to discriminate changed samples deserves careful examination. The outlier threshold used in this study for outlier identification was 5 for all land cover types except Water body whose outlier threshold is 3. These outlier thresholds were chosen with observations of outlier score distributions of samples (Figure 2). The total number of identified outlier samples based on the threshold is less than 5% of the original samples. Due to the lack of reference data in 2001, the removed samples were not verified if they indeed were all changed samples. It is likely that for the study region which is agricultural production dominant, the removed samples may contain some unchanged samples, which were not representative anymore of the classes due to noises such as cloud/cloud shadow, etc. It is advisable to analyze the outlier score distribution of samples and then to determine the threshold for outlier identification. In a general sense, a large threshold removes fewer outliers, and a small threshold removes more. It is safer to use a small threshold as changed samples are more likely identified as outliers. Although a small threshold would remove some unchanged samples as well, it would not have much impact on the modelling result if there is still a sufficient number of remaining samples for modelling. The goal of the threshold determination exercise is to be able to remove all changed samples while keep unchanged ones. In this regard, although the outlier score distribution shown in Figure 2 gives us some ideas what thresholds should be for identifying outlier samples, further study of the outlier scores’ distribution and characteristics of samples with various features is truly needed.

Second, knowledge (sample) transferring is conducted between adjacent years. The two consecutive years (2000 and 2001) had a similar climatic condition: both were drought years. It is worth to study if the proposed method is still useful for adjacent years with different climates or for non-adjacent years with a similar environmental condition. If yes, in what degree can it be effective? If climate patterns of two study years, of source-domain and target-domain are different, e.g., one drought and one damp, they will have different phenology patterns of vegetation, and sample transfer may be ineffective; if similar environmental conditions are met, then the sample transfer method may work well. Therefore, one critical condition for knowledge transfer from source domain to target domain is how similar the environmental conditions are that account for the similar phenology patterns of vegetation.

Third, one drawback of this method is that we are not able to identify the cause of the “outliering”, either because of changes of land cover type or due to other reasons such as data noise. Removing the outlier samples resulting from the two causes is desirable. Fourth, it is likely that some changed samples may remain in the transferred samples while some unchanged samples are removed. One reason is the dataset used in this study is 10-day composites. Because actual dates of different pixels can be different the phenology can be different, especially in the vigorous growing season. According to the outlier identification method with a cut-off threshold, some unchanged samples would be wrongly removed while changed samples may remain. Therefore the impact on sample data transferring of the characteristics of RF’s outlier score of various features at different timeframes of time-series EO data deserves further focused study.

## 6. Conclusions

In this study we tackled two important issues regarding how to efficiently use a large volume of time-series EO data and knowledge transferring of sample data to applications of unavailable samples for supervised land cover classification modelling. To achieve these goals we investigated Random Forests’ two valuable features of variable importance and outlier identification with time-series MODIS data in the application of land cover classification at a regional level. Using a case study, we conducted Random Forests modelling with CCRS time series 10-day clear-sky MODIS composites to explore RF variable importance feature for analyzing optimal variable combinations of band and its seasonal information for efficient and effective land cover classification. We also explored Random Forests’ outlier feature for transferring samples to its adjacent year when no samples were available for supervised classification modelling. The results of this study demonstrate promise of the developed methods for solving the two important issues of optimal subset selection and sample data transfer. Based on our study, we make the following conclusions:

(a) For utilization of time-series MODIS data or other time-series EO datasets for applications such as land cover mapping, there are possible hundreds of variables and their combinations available. In general, more variables from a longer time period would produce more accurate results; however, there is a plateau in the accuracy of land cover classification after a certain number of variables are used. In this study, there are 147 variables in the dataset and using about half of the variables reaches the plateau (Kappa of ~0.83). Information produced from the study suggests that in the study region, MODIS composite images from the months of April, July and August, and bands of 1, 2 and 3 contribute the most to the accuracy of land cover classification. In addition, based on the results of the experiment runs, it can be expected that using the early season (from April to August) of the time-series MODIS composites could generate a land cover map similar in accuracy as using the full dataset.
(b) Random Forests modelling produces a variable importance score to measure the variable’s contribution to its modelling. The case study of land cover classification with time-series MODIS dataset demonstrates that the Random Forests’ variable importance could be utilized to efficiently select an optimal subset from the time-series EO data for improving computation efficiency while reaching the highest possible accuracy. This method could be beneficial to other time-series EO dataset analysis, for example, the coming time-series Sentinel 2–3 dataset.
(c) Ground truth or samples are essential for supervised classification modelling. One common problem of supervised modelling of time-series EO data analysis is the lack of sample data for model training and validation. Knowledge or sample data transferring from one timeframe to another provides a way to tackle this critical issue. The proposed simple but effective method used in this study could be practical for applications where ground truth is lacking but is available at an adjacent timeframe with similar environmental conditions, for example in the case of historical dataset. The proposed method also has large economic benefit as field work for data sampling is usually costly. Random Forests’ outlier identification feature is found useful for achieving this goal. Studies focused on improving the understanding of the outlier characteristics of ground features for sample transfer for supervised land cover classification modelling should get more attention in the future.

## Figures and Tables

**Table 1:** Results of experimental runs. OOB: Out of Bag.

Subset Data	Number of Months	Number of Bands	Number of Variables	Kappa	OOB Error (%)
1	4	6	72	0.816	16.344
2	4	7	84	0.817	16.268
3	5	5	75	0.817	16.285
4	5	6	90	0.821	15.936
5	5	7	105	0.819	16.118
6	6	4	72	0.822	15.825
7	6	5	90	0.823	15.704
8	6	6	108	0.825	15.537
9	4 (April, May, June, July)	7	84	0.814	16.525
